# Amylopectin xerogel with onion based sulfur nitrogen doped carbon quantum dots as a chemosensor for chromium and biosensor for microbial spoilage in tomatoes

**DOI:** 10.1038/s41598-025-19875-x

**Published:** 2025-09-23

**Authors:** Hebat-Allah S. Tohamy

**Affiliations:** Cellulose and Paper Department, National Research Centre, 33 El Bohouth Str., P.O. 12622, Dokki, Giza, Egypt

**Keywords:** Amylopectin, Poly(N-isopropylacrylamide), Flavonoids, Colored chemosensor, Biosensor, pH-sensing, Food packaging, Environmental sciences, Chemistry, Materials science

## Abstract

This study presents the development of a multifunctional, biodegradable xerogel film based on amylopectin and poly(N-isopropylacrylamide) (poly(NIPAm)) incorporating sulfur and nitrogen-doped carbon quantum dots (S, N–CQDs) derived from red onion peels (ROP). The formation mechanism and stability of the composite film were investigated using DFT calculations, revealing enhanced interactions and stability in the S, N–CQDs-containing film (amylopectin-S, N–CQDs15). FTIR and SEM analyses confirmed the successful incorporation of S, N–CQDs and revealed a tighter pore structure in the composite film, leading to increased surface area. The amylopectin-S, N–CQDs15 film exhibited significantly improved antibacterial activity, with inhibition rates of 95.25% against *Escherichia coli*, 99.12% against *Staphylococcus aureus*, and 99.23% against *Candida albicans*. These findings were supported by molecular docking studies indicating strong binding affinities. Furthermore, the film demonstrated its potential as a smart sensor through distinct fluorescence responses to these microorganisms: it showed mixed green and red fluorescence with *E. coli*, blue dots with *S. aureus*, and a change from large red regions to numerous green dots with *C. albicans*. The film also exhibited a fluorescence shift from red to blue upon exposure to Cr(VI). Notably, the film displayed pH-responsive color transitions relevant to monitoring tomato spoilage. These findings highlight the potential of this bio-based composite film, prepared from a waste resource, as a sustainable and effective solution for active food packaging, offering antimicrobial properties and detection of spoilage and contamination.

## Introduction

The pervasive global challenge of food spoilage and waste constitutes a significant economic and environmental burden, particularly concerning highly perishable commodities like tomatoes^1^. This issue leads to immense financial losses for producers and consumers while contributing to greenhouse gas emissions from landfills. A primary driver of this spoilage is microbial contamination, posing a direct threat to public health. Pathogens such as *Escherichia coli*, *Staphylococcus aureus*, and *Candida albicans* can proliferate rapidly, causing foodborne illnesses that range from mild to life-threatening. Traditional methods for detecting these contaminants are often slow, labor-intensive, and require specialized laboratory equipment, making them impractical for real-time, on-site assessment. Consequently, there is an urgent and critical need for the development of rapid, sensitive, and accessible detection technologies to ensure food safety and mitigate waste at its source^2–6^. While often overlooked compared to microbial contamination, heavy metal contamination, particularly by chromium (Cr), presents a serious and distinct threat to food safety. Unlike microbial spoilage, which involves visible signs like mold or fermentation, heavy metal contamination can be silent and insidious, as it does not inherently cause visible spoilage or changes in food appearance. The escalating global concerns surrounding heavy metal contamination, particularly chromium, pose a significant threat to environmental health and ecological balance worldwide^7,8^. This issue is further compounded by the substantial economic and health ramifications of food spoilage, a challenge that can be exacerbated by the presence of such contaminants through various pathways. For instance, chromium can leach into canned goods like tomatoes from the protective lining of the can itself, particularly if the can is damaged or improperly stored^9,10^. Furthermore, agricultural land can become contaminated with chromium through industrial waste, irrigation with polluted water, or the use of certain fertilizers, leading to its uptake by crops^11,12^. The convergence of these two critical problems, the introduction of heavy metals into our food supply and the subsequent spoilage, demands urgent attention and comprehensive strategies to safeguard both our ecosystems and the integrity of our food supply chains^13^. Addressing these intertwined challenges necessitates the development and deployment of rapid, cost-effective, and reliable sensing technologies. Traditional analytical methods, such as Atomic Absorption Spectroscopy (AAS) or Inductively Coupled Plasma Mass Spectrometry (ICP-MS), while highly accurate, present several drawbacks that hinder widespread and timely monitoring^14–16^. These techniques often involve complex sample preparation procedures, including digestion and extraction, which can be labor-intensive and time-consuming, potentially taking hours or even days to yield results. The high cost associated with the specialized equipment, the need for controlled laboratory environments, and the requirement for trained technicians further limit their applicability for routine and on-site analysis^3^. Moreover, the destructive nature of some of these methods means that the sample cannot be used for further analysis or consumption^17^. Given the complex challenges of microbial and heavy metal contamination, a paradigm shift is needed from passive food containment to a proactive, intelligent approach. This shift is embodied by smart packaging and active packaging technologies. Unlike traditional packaging that simply protects food from the external environment, these advanced systems actively monitor and preserve product quality and safety throughout the supply chain. Active packaging incorporates components that release or absorb substances to improve food quality, such as oxygen scavengers or antimicrobial agents. Smart packaging, on the other hand, includes features that sense and communicate information about the product’s condition, such as heavy metal contamination, gas levels, or the presence of pathogens^2–4,13^. The recognition element selectively binds to or interacts with a target analyte (like a bacterium or a heavy metal ion). This interaction then triggers a physical or chemical change that the transducer converts into a measurable signal, such as a change in color, fluorescence, or electrical current. This seamless integration allows for the rapid, on-site detection of contaminants, offering a powerful solution to overcome the limitations of conventional laboratory analysis and paving the way for real-time food safety monitoring^3,4,18^.

In the pursuit of more efficient and accessible sensing solutions, nanomaterials have emerged as promising candidates, offering unique properties that can overcome the limitations of conventional techniques^19^. Among these, carbon quantum dots (CQDs) have garnered significant attention due to their remarkable optical characteristics, including strong fluorescence emission with tunable wavelengths, excellent photostability, and low toxicity^4,20–23^. Furthermore, their relatively simple and cost-effective synthesis, coupled with the potential for facile surface functionalization with various chemical groups, makes them highly versatile for the development of selective and sensitive sensors for a wide range of analytes, including heavy metal ions like chromium^13^. The inherent advantages of CQDs position them as a powerful platform for creating the next generation of rapid, cost-effective, and reliable sensing technologies for both environmental monitoring and food safety applications. One major challenge in developing sustainable materials is finding a viable source for raw materials. This study addresses that challenge through the concept of waste valorization, which involves transforming agricultural or industrial waste into valuable products. Red onion peels (ROP), a common and often discarded byproduct of the food industry, are rich in carbon and other organic compounds, making them an ideal, low-cost precursor for synthesizing advanced nanomaterials. In this work, we demonstrate the successful valorization of ROP by using it to prepare sulfur and nitrogen-doped carbon quantum dots (S, N–CQDs). This approach not only provides a sustainable and economical alternative to conventional sources but also helps to mitigate agricultural waste. By incorporating these waste-derived CQDs into a biodegradable amylopectin matrix, we create a highly effective and environmentally friendly composite, transforming a a useless byproduct into a valuable component for intelligent food packaging. It can indicate food spoilage through subtle yet discernible color variations, essentially acting as an integrated freshness indicator. Moreover, their unique optical properties enable the detection of early, invisible indicators of microbial proliferation. This dual capability holds the potential to significantly reduce food waste by providing timely information about food quality while simultaneously promoting sustainability by transforming discarded materials into advanced packaging solutions. Although still in the early stages of development, this concept of embedding responsive nanomaterials within food packaging signifies a substantial advancement towards a more resource-efficient and environmentally responsible food management system. The natural pigments present in onion peel waste exhibit a striking sensitivity to changes in acidity and alkalinity. These compounds, known as flavonoids, typically present a yellow color in acidic conditions, shift to orange as neutrality is approached, and often turn red in alkaline environments^1,24,25^. This color-changing behavior is attributed to the different structural forms, or isomers, that these flavonoids can adopt, with their prevalence being influenced by the surrounding pH, subtly altering the overall color^26,27^. However, directly adding S, N–CQDs to food packaging faces hurdles. Their extremely small size and high surface energy cause them to clump together and settle, which can lead to uneven distribution within the packaging material and potentially compromise its intended functions^28^. Furthermore, managing and processing these powdered CQDs can be intricate, suggesting the need for alternative approaches like embedding them within a protective polymer or creating stable liquid forms to ensure their effective and safe use in food packaging^2,29^. To address these handling limitations and enhance the practical applicability of the onion-based S, N–CQDs, we focused on the fabrication of biodegradable hydrogel films. These films offer a stable and easily processable matrix for the S, N–CQDs, potentially enabling their integration into food packaging materials for real-time monitoring of both environmental contaminants and food quality. In this study, we report the facile synthesis of fluorescent/colored S, N–CQDs derived from ROP and their subsequent incorporation into an amylopectin-based hydrogel matrix. This biodegradable film was then investigated for its dual functionality as a visual chemosensor for Cr(VI) ions and as a biosensor for detecting microbial spoilage in strawberries, offering a promising avenue for enhancing food safety and reducing food waste. Complementary computational studies were also conducted to elucidate the underlying mechanisms governing the interaction of the CQDs with the target analytes, providing crucial insights into the observed sensing behavior.

Given its affordability and water-loving nature as a glucose-based biopolymer, starch presents itself as a compelling starting material for creating hydrogels^30^. Starch is composed of two main components: a linear polymer called amylose, typically with a molecular weight ranging from 200,000 to 2,000,000, and a branched polymer known as amylopectin, which can reach extremely high molecular weights up to 400 million. The irregular, branched architecture of amylopectin, along with its numerous hydroxyl (-OH) groups, facilitates its dispersion in water to form colloidal solutions, making it particularly useful in hydrogel formation^31,32^. Amylopectin is one of the two major polysaccharides that constitute starch, the primary energy storage carbohydrate in plants, with the other being amylose^33^. Typically, amylopectin makes up a larger proportion of starch, generally ranging from 70 to 80%, although this ratio can vary depending on the botanical source^34^. Structurally, amylopectin is a highly branched polymer of α-D-glucose units. The glucose units are primarily linked by α-(1→4) glycosidic bonds, forming short linear chains^32^. However, another widely adopted approach involves creating interpenetrating networks by blending starch with other water-absorbing polymers, including poly(N-isopropylacrylamide) (PNIPAm)^35^. PNIPAm serves as the primary component in the single-network hydrogels examined in this research. These hydrogels demonstrated a notably swift response to alterations in pH^36,37^. The selection of PNIPAm was deliberate, as this well-researched and widely utilized polymer exhibits a sensitivity to pH changes, making it particularly promising for developing responsive sensing elements within food packaging applications^38,39^.

Tomatoes spoilage presents a significant concern within the food supply chain, mirroring challenges. This deterioration leads to considerable economic losses for producers and retailers, as well as contributing to the growing issue of food waste^40,41^. Tomatoes are vulnerable to rapid colonization by spoilage microorganisms, including molds and bacteria, which break down the fruit’s structure and flavor^42^. Factors throughout the supply chain, from the initial harvesting and handling to storage conditions and transportation, can significantly influence and accelerate the rate of spoilage^43,44^. To combat these issues and extend the shelf life of tomatoes various preservation techniques are currently employed, such as refrigeration and modified atmosphere packaging^45,46^. However, in line with the increasing consumer preference for natural and sustainable food preservation methods, innovative alternatives are being explored. One promising approach involves the application of edible films and coatings based on materials like the amylopectin-rich hydrogels we’ve discussed. These hydrogel films, derived from the branched structure of amylopectin, offer a sustainable alternative to conventional plastic packaging^47^. When applied to strawberries, these amylopectin-based hydrogels can slow down the metabolic processes that lead to spoilage^48^. Furthermore, the inherent properties of the hydrogel matrix could potentially be enhanced with the incorporation of natural antimicrobial agents such as CQDs, further inhibiting the growth of spoilage-causing microorganisms on the surface of the strawberries^49^. This research introduces a significant advancement in sustainable food packaging technology. We have successfully developed a novel, multifunctional xerogel film by incorporating sulfur and nitrogen-doped carbon quantum dots (S, N–CQDs) derived from a waste product, red onion peels, into an amylopectin matrix. This approach offers a dual-action system that goes beyond simple preservation. The resulting amylopectin-S, N–CQD xerogel film is a sustainable and effective solution for combating food spoilage and contamination. It provides a powerful antimicrobial barrier to inhibit the growth of common pathogens like *E. coli*, *S. aureus*, and *C. albicans*. Simultaneously, it functions as a real-time biosensor, providing a distinct visual signal for both microbial spoilage and the presence of toxic Cr(VI). To demonstrate the real-world utility of this intelligent food packaging system, a comprehensive verification process would be essential. The prepared amylopectin-S, N-CQDs15 films would be applied to fresh and contaminated tomato samples. These films’ ability to provide a rapid, on-site visual indication of spoilage and contamination would be monitored over time. This would move the findings from a controlled lab environment to a practical application, confirming the sensor’s potential to enhance food safety and reduce waste in a consumer or retail setting. This dual capability, derived from a low-cost, bio-based material, positions our composite film as a promising solution for the next generation of smart food packaging, enhancing food safety and reducing waste. Table 1 has made a comparative overview of the biological characteristics of previously formulated sensors in relation to biological attributes of the prepared xerogels synthesized in the current study.

**Table 1:** A comparative overview of the biological characteristics of previously formulated sensors in relation to biological attributes of the prepared xerogels synthesized in the current study.

Sensor	CQDs source	Antibacterial activity	Drawbacks	Advantages	Ref.
Amylopectin- S, N–CQDs15	Red onion peels	The amylopectin-S, N–CQDs15 film showed inhibition rates of 95.25% against *Escherichia coli*, 99.12% against *Staphylococcus aureus*, and 99.23% against *Candida Albicans* by CFU method.	Lack of mechanical strength due to the xerogel nature.	Detection of Cr(VI) and bacteria by a naked-eye response.	This study
CMC-Betalains-N–CQD composite	Beet root peels	The antibacterial activity against *Escherichia coli*, *Staphylococcus aureus*, and *Candida. Conversely* were evidenced by inhibition zones of 20, 21, and 21 mm, respectively.	It solubilizes easily because the main polymer is CMC.	It offers a dual-mode detection system using both naked-eye color changes and luminescence. This versatile platform can be applied to complex samples like strawberries and also possesses shape-memory properties.	^3^
HEC-S, N–CDs2	Red onion peels	HEC-S, N–CDs2 exhibited antibacterial activity by CFU method against *Salmonella enterica* equal 70%.	The HEC is commercial not recycled from any agrowaste.	Excellent mechanical and antimicrobial activity. In addition, it can detect meat spoilage from a distane after packaging on the top of the container which means high efficiency.	^4^
CMC–N-fullerene–AMPS hydrogel		CMC–N-fullerene–AMPS hydrogel displayed a substantial inhibition zone of 22 and 24 mm against *Escherichia coli* and *Staphylococcus aureus*, respectively.	It’s just fluorescent detection for spoilage. There is no naked-eye detection.	It offers a “turn-on” fluorescence response for bacterial detection, enabling a clear fluorescent signal. The research also includes advanced computational analysis using Density Functional Theory (DFT) and molecular docking to understand the sensor’s mechanism at a molecular level.	^18^
CMC-SN–CDs	Red onion peels	The CMC-SN–CDs displayed antibacterial activity against *Escherichia coli* and *Staphylococcus aureus* by CFU method equal to 20.00 and 32.15%, respectively.	It’s just detected the microbial spoilage and there is no detection for heavy metals pollution.	The developed biosensors enable naked-eye visual detection of tomato spoilage with good mechanical properties.	^1^
HPMC-MCDs	Magnetite CQDs from sugarcane bagasse	The HPMC-MCDs showed binding with *Salmonella enterica* and *B. cereus* protein with bond length ~ 1.67 and 2.83 A°, respectively, which means the high binding between HPMC-MCDs and *Salmonella enterica*.	Magnetite can generate reactive oxygen species, posing a safety risk if the nanoparticles were to migrate into the packaged beef.	The key advantage is the novelty of creating a naked-eye sensor using magnetite carbon dots. Before this work, no one had developed a visual, color-based sensor from magnetite, which typically only has magnetic and fluorescence properties. This makes it a pioneering and highly accessible detection method.	^2^
CM-Hemi@Ca-N–CDs	Sugarcane bagasse	CM-Hemi@Ca-N–CDs exhibited antibacterial activity against *Escherichia coli*, *Staphylococcus aureus*, and *Candida equal* 13, 15, and 11 mm, respectively.	Its dected microbial spoilage by fluorescent. There is no naked-eye detection.	Its 100% biodegradable and recycled from agrowastes.	^50^

## Materials and methods

### Materials

Red onion peels waste (OPW), sourced from a local Egyptian kitchen, was employed to synthesize SN–CDs. N-Isopropylacrylamide (NIPAm), N,N’-methylenebis(acrylamide) (BAA), sodium methacrylate (SMA), ammonium persulfate (APS), N,N, N’, N’-tetramethylethylenediamine (TEMED), and amylopectin (‘‘Starch from corn’’, product nr. S9679) Obtained from Sigma-Aldrich and used without further purification.

### Preparation of sulfur, nitrogen doped carbon dots from red onion peels

For the production of S, N–CQDs utilizing ROP, a consistent mixture was formulated using 4 g ROP, 9.33 g NaOH, 9.33 g thiourea, and 100 mL water. The inclusion of thiourea aimed to enhance the solubility of the cellulosic material present in the OPW. This resulting mixture was then treated through a series of physical steps: initial freezing, followed by sonication, and concluding with 7 min of microwave irradiation at 700 W^51^.

### Amylopectin xerogel preparation

Polymerization was conducted at a temperature of 60 °C. The initial components NIPAm (0.5 g), BAA (0.0109 g), SMA (0.015 g), TEMED (0.015 g), and amylopectin (4.7890 g) were thoroughly stirred. Following stirring, 15% S, N–CQDs (w/w) was added with continues stirring. The polymerization was initiated by the addition of a 1% aqueous solution of APS. The reaction was allowed to proceed for 24 h, at which point it was deemed complete. This xerogel was denoted as amylopectin-S, N–CQDs15. The reference xerogel was prepared without S, N–CQDs and denoted as amylopectin- S, N–CQDs0 (Table 2).

**Table 2:** Amylopectin xerogel components.

Components	Amylopectin-S, *N*–CQDs0	Amylopectin-S, *N*–CQDs15
NIPAm (g)	0.5	0.5
BAA (g)	0.0109	0.0109
SMA (g)	0.015	0.015
Amylopectin (g)	4.7890	4.7890
S, N–CQDs (%)	0	15
APS (%)	1	1
TEMED (g)	0.015	0.015

### Amylopectin-S, N–CQDs xerogel film application to monitor and preserve tomatoes

The fresh tomatoes were purchased from the Egyptian local shop of Cairo, Egypt. The tomatoes were packed indirectly with films made of amylopectin-S, N–CQDs0 (non-colorimetric film) and amylopectin-S, N–CQDs15 (colorimetric film) separately. The tomatoes were subsequently stored in at 25 °C $\:\pm\:$ 1 for duration of 10 days. A smartphone camera was used to examine and photograph the color.

### Characterization

#### Determination of the pH-sensitivity of intelligent amylopectin-S, N–CQDs15 xerogels

The pH sensitivity of the smart film was evaluated by immersing it for 30 s in buffer solutions at pH 3 (acidic) and pH 12 (basic), following the procedure outlined by Tohamy et al. Color variations were documented with a smartphone^1^.

#### Morphological observation

Microstructural analysis was performed using a Quanta/250-FEG scanning electron microscope (Thermo Fisher Scientific, Waltham, MA, USA) to obtain SEM images. 

#### Fluorescence microscope

Fluorescence microscopy was performed using a Jasco FP-6500 spectrofluorometer (made in Japan) with a 150-watt xenon arc lamp.

#### Fourier-transform infrared (FTIR) spectra

FTIR spectra were recorded using a Mattson 5000 spectrometer (Unicam, United Kingdom) with KBr pellets. The crystallinity index (LOI) and mean hydrogen bond strength (MHBS) were calculated using the following equations:

1 $${\text{LOI}} = \frac{{A_{{1425}} }}{{A_{{900}} }}$$

2 $${\text{MHBS}} = \frac{{A_{{OH}} }}{{A_{{CH}} }}$$

where A_1425_ and A_900_ refer to the FTIR absorbance at 1425 and 900 cm^− 1^, respectively. In addition, A_OH_ and A_CH_ refer to the FTIR absorbance of the OH and CH peaks, respectively^52–59^.

#### DFT calculations

Calculations based on density functional theory (DFT) were carried out using the Gaussian 09 W software package, employing the B3LYP hybrid functional and the 6-31G(d) basis set. Geometry optimization was performed using the Berny algorithm. Several parameters, including optimized geometries and ground state energies, were explored through these DFT calculations, including total energy (E_T_), the energy of the highest occupied MO E_HOMO_, the energy of the lowest unoccupied MO E_LUMO_, the energy gap (E_g_), the dipole moment (µ), the absolute hardness (η), the absolute softness (σ), the chemical softness (S), and the additional electronic charge (ΔN_max_)^60,61^.

3 $$\:{E}_{gap}=({E}_{LUMO}-{E}_{HOMO})$$

4 $$\:{\upeta\:}=\frac{({E}_{LUMO}+\:{E}_{HOMO})\:\:}{2}\:$$

5 $$\:{\upsigma\:}=\frac{1\:\:}{{\upeta\:}}$$

6 $$\:\text{S}=\frac{1\:\:}{2{\upeta\:}}$$

7 $$\:{\Delta\:}{N}_{max}=\frac{-\text{P}\text{i}\:\:}{{\upeta\:}}$$

## Results and discussion

### Mechanism of amylopectin-S, N–CQDs xerogel film formation with DFT calculations

**Scheme 1:**
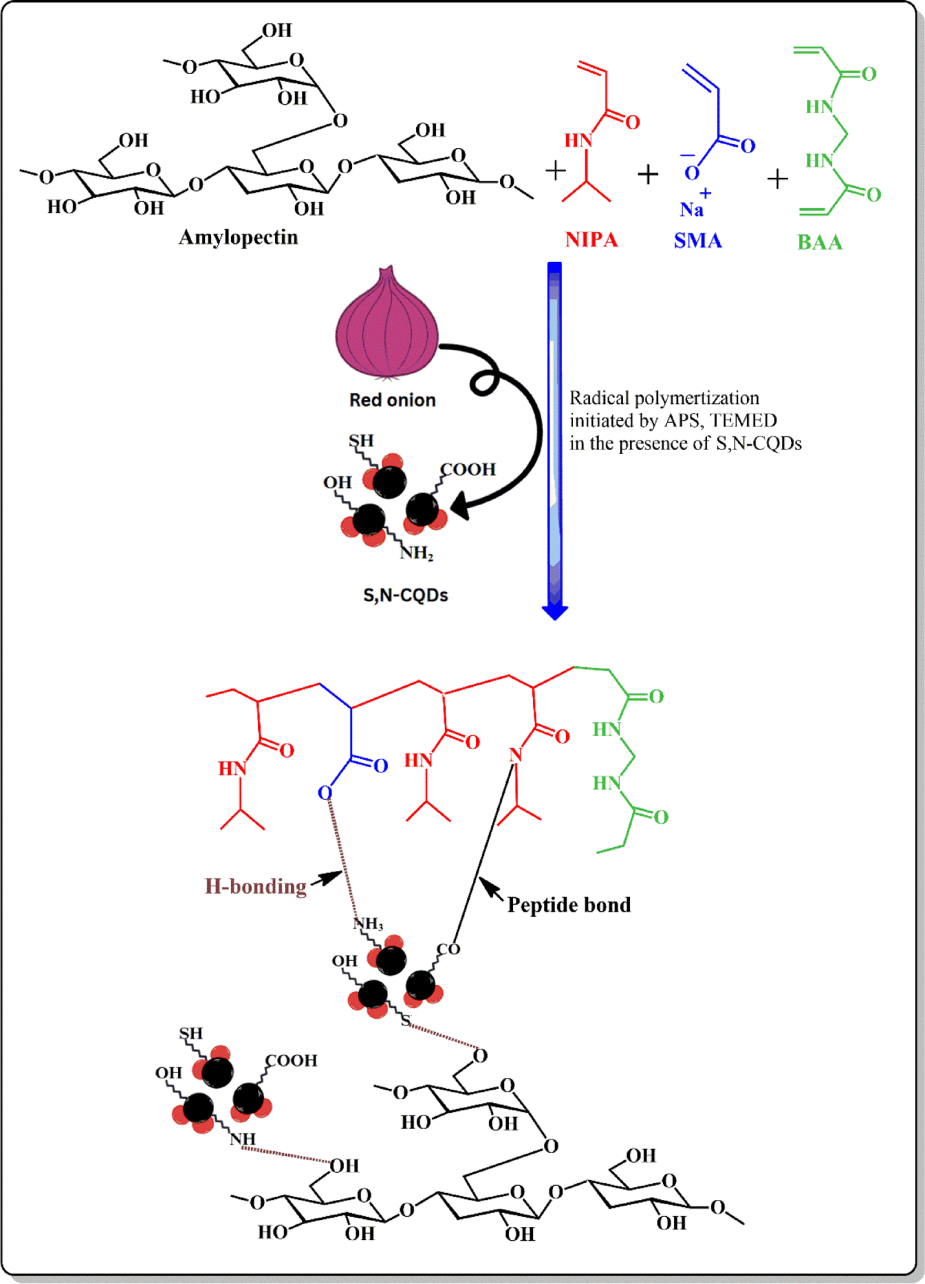
Hydrogen bonding and amidation bonding (peptide bond) between amylopectin, Poly(NIPAm) and S, N–CQDs for synthesizing amylopectine- S, N–CQDs xerogel.

The formation of amylopectin-S, N–CQDs xerogel film involves a complex network of intermolecular interactions between the functional groups of amylopectin, Poly(NIPAm) and S, N–CQDs (Scheme 1). These interactions include:

By co-polymerizing NIPAm and SMA double bonds in the presence of dispersed amylopectin. APS and TEMED were employed as radical co-initiators, while BAA was added to chemically crosslink the poly(NIPAm-co-SMA) chains. Previous work informed the choice of SMA content to ensure high pH-sensitivity^31^. The OH groups from amylopectin can form hydrogen bonds with each SH and NH_2_ groups of S, N–CQDs. Additionally, the carboxylate groups in the polymer network (from SMA) might interact electrostatically with any positively charged regions on the surface of the S, N-CQDs (due to protonation of nitrogen-containing groups). Moreover, prolonged heating can sometimes drive the reaction forward by overcoming the activation energy, especially in the presence of dehydrating conditions (to remove water formed). The reaction is at 60 °C, which might provide enough energy for some slow reaction. As a result help in amidation reaction between the COOH group of S, N–CQDs and NH of the amide group for BAA. These intermolecular interactions contribute to the cross-linking and network formation within the hydrogel film, leading to its unique properties and applications.

**Fig. 1:**
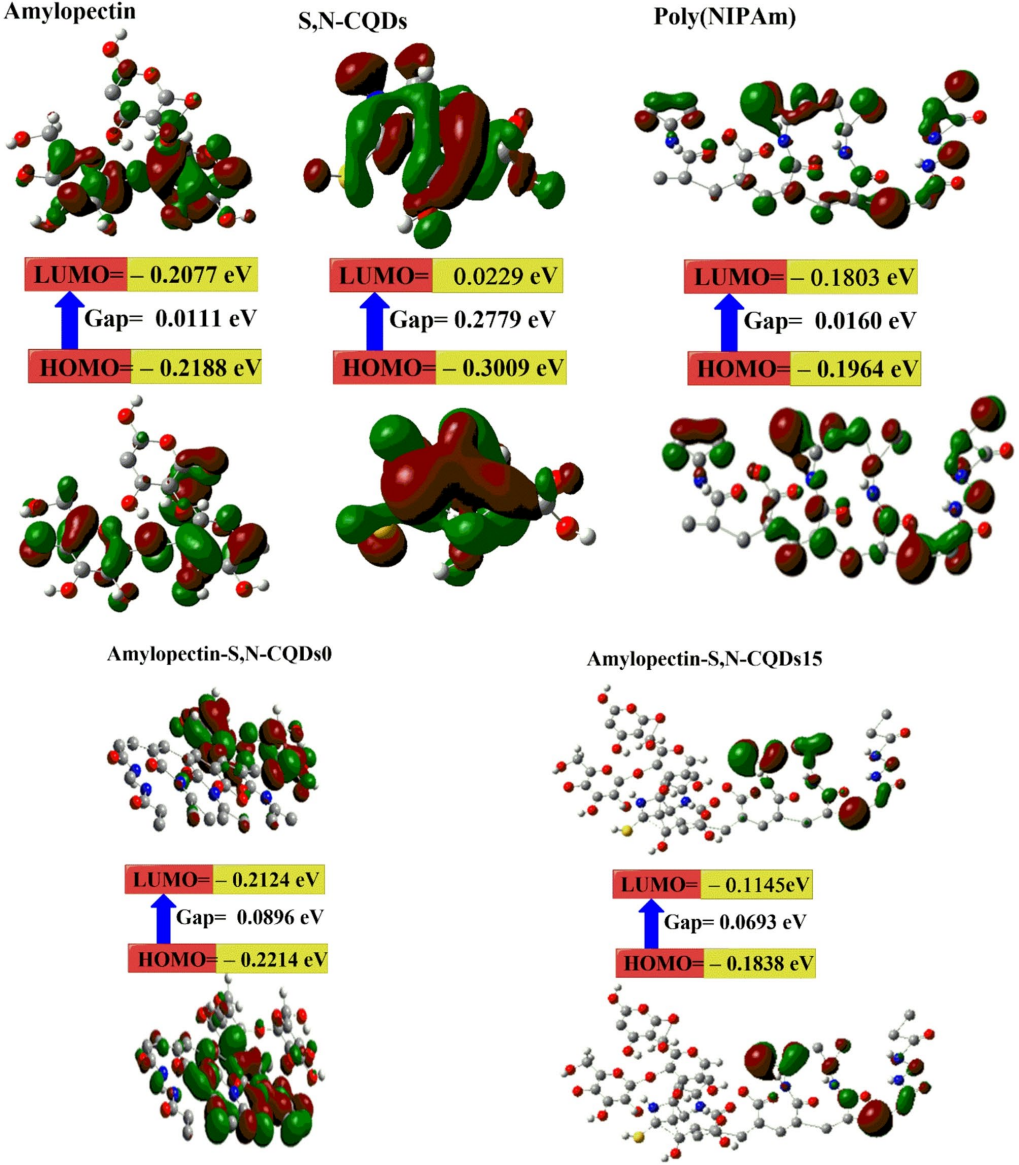
The gap energies (HOMO–LUMO) (eV) were calculated for the hydrogels using DFT B3LYP/6–31G (d), as was the molecular orbital interaction between amylopectin, S,N–CQDs, Poly(NIPAm), amylopectine-S, N–CQDs0 and Amylopectin-S, N–CQDs15.

DFT calculations were employed to study the stability of the amylopectin, S,N–CQDs, Poly(NIPAm), amylopectin-S, N–CQDs0 and Amylopectin-S, N–CQDs15 (Table 3; Fig. 1). The higher µ of amylopectin-S, N–CQDs0 compared to amylopectin-S, N–CQDs15 The difference in dipole moments between amylopectin-S, N-CQDs0 (9.55 Debye) and amylopectin-S, N-CQDs15 (6.94 Debye), as indicated by DFT calculations, can be attributed to the influence of S, N-CQDs on the overall charge distribution within the composite material. Amylopectin and poly(NIPAm) possess inherent dipole moments due to the electronegativity differences between their constituent atoms. However, the introduction of S, N–CQDs, with their unique electronic properties and potential for complex interactions, can significantly alter this initial charge distribution. One key factor is the interaction between S, N–CQDs and the regions of high charge density within the amylopectin and poly(NIPAm) molecules. These interactions, which may include hydrogen bonding, van der Waals forces, and electrostatic interactions, can effectively “neutralize” some of the existing dipoles, leading to a decrease in the overall dipole moment of the amylopectin-S, N-CQDs15 composite. Furthermore, the addition of S, N–CQDs may induce conformational changes in the polymer chains, resulting in a more symmetrical arrangement of charges and a greater cancellation of individual dipole moments. For a food packaging sensor designed to detect bacteria, a low dipole moment is generally more desirable. A low dipole moment can contribute to a more stable, selective, and reliable sensor, which are critical requirements for this application. While a high dipole moment might enhance the interaction with bacteria, it could also lead to increased interference, instability, and irreversible binding.

The lower E_g_ of amylopectin-S, N-CQDs15 (0.0693 eV) compared to amylopectin-S, N-CQDs0 (0.896 eV) indicates a strong chemical interaction between amylopectin, S,N–CQDs, and poly(NIPAm) in the amylopectin-S, N-CQDs15 composite^29,49^. Moreover, the lower E_T_ of amylopectin-S, N-CQDs15 (– 4455.68 au) compared to amylopectin-S, N-CQDs0 (– 3567.38 au) indicates a more stable amylopectin-S, N-CQDs15 composite structure^13,60^. Additionally, the Pi is negative for amylopectin-S, N-CQDs15 (i.e., − 0.1461 eV) which means stability^1,50,60^.

The high ɳ of amylopectin-S, N-CQDs15 (– 0.1491 eV) enhance the resistance to deformation, scratching, or indentation, offers advantages such as enhanced durability and abrasion resistance, which are crucial for maintaining sensor functionality throughout the food’s shelf life^60,62^.

**Table 3:** The quantum chemical parameters of amylopectin, S,N–CQDs, Poly(NIPAm), amylopectin-S, N–CQDs0 and amylopectin-S, N–CQDs15.

DFT	Amylopectin	S, *N*–CQDs	Poly(NIPAm)	Amylopectin-S, *N*–CQDs0	Amylopectin-S, *N*–CQDs15
E_LUMO_ (eV)	–0.2077	– 0.0229	–0.1803	–0.2124	–0.1145
E_HOMO_ (eV)	–0.2188	– 0.3009	–0.1964	–0.2214	–0.1838
E_g_ (eV)	0.0111	0.2779	0.0160	0.0896	0.0693
E_T_ (au)	–1707.50	– 939.24	–1855.28	–3567.38	–4455.68
µ (Debye)	11.18	2.640	–5.03	9.55	6.94
ɳ (eV)	–0.21332	– 0.1619	–0.1883	–0.2169	–0.1491
σ (eV)	–4.68779	– 6.1760	–5.3081	–4.60	–6.70
Pi (eV)	–0.2133	–0.1619	–0.1883	0.2169	–0.1461

### Fourier transform infrared spectroscopy (FTIR) spectra

**Fig. 2:**
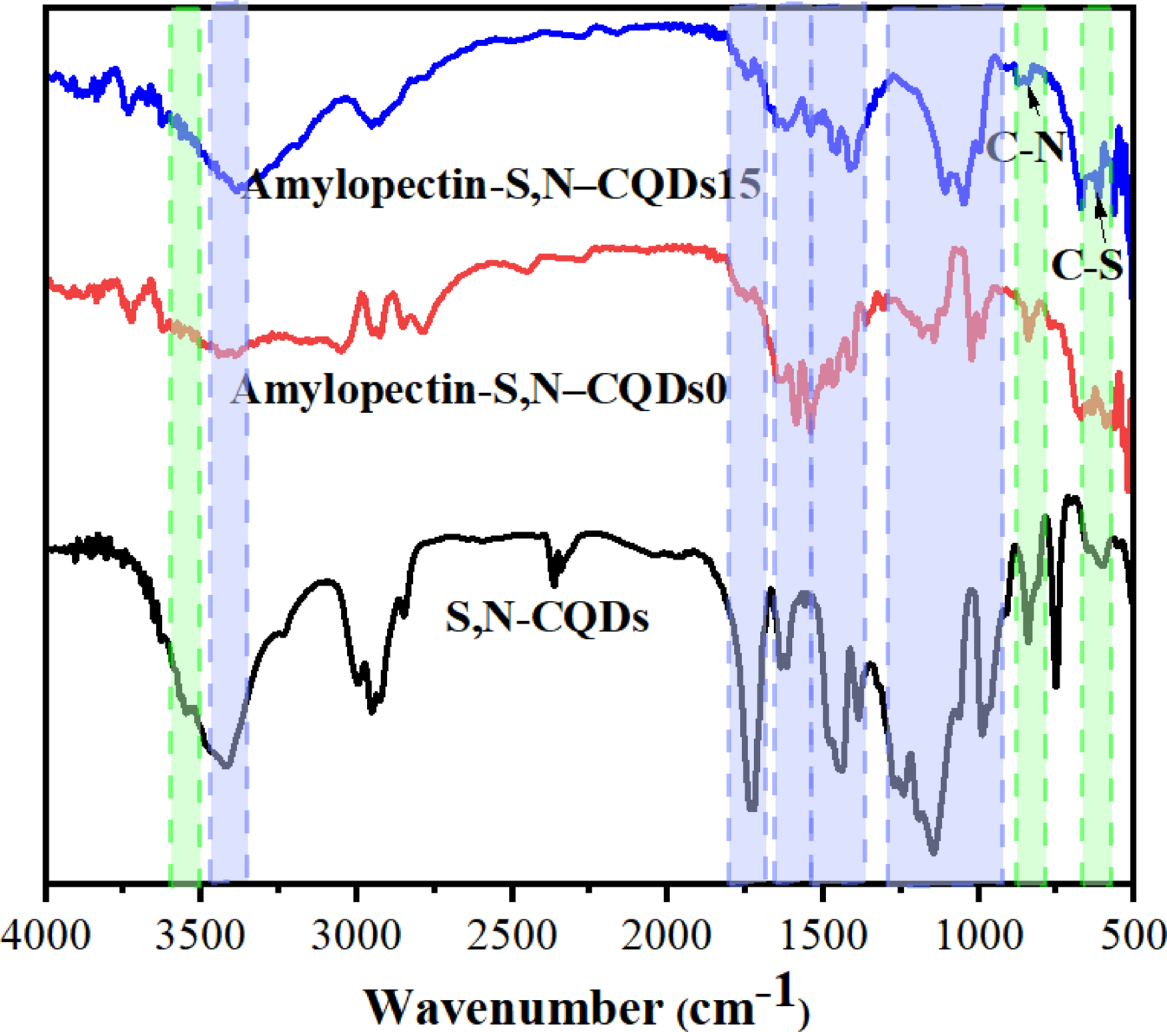
FTIR spectra of S, N–CQDs, amylopectin-S, N–CQDs0 and amylopectin-S, N–CQDs15.

The S, N–CQDs showed peaks at 3552 cm^–1^, 3423 cm^–1^, 2954 cm^–1^, 1733 cm^–1^, 1617 cm^–1^, 1440 cm^–1^, 1384 cm^–1^, 1247 cm^–1^, 1139 cm^–1^, 989 cm^–1^, 884 cm^–1^, and 750 cm^–1^ related to N–H, O–H, C–H, C = O, amide I, amide II, C = C, O–C = O, C–O–C, C–O, C–N, and C–S, respectively. The identification of N–H and C–N spectral features demonstrates the successful introduction of nitrogen into the structure of the S, N–CQDs (Fig. 2). Correspondingly, the detection of C–S and S–H peaks in the spectra indicates the effective doping of sulfur within the S, N–CQDs (Fig. 4). The amylopectin-S, N–CQDs0 showed peaks at 3442 cm^–1^, 1540 & 1469 cm^–1^, 1143 cm^–1^, and 1024 cm^–1^ related to O–H, angular deformation of C–H, C–O ether bond, C–O alcohol bond because of the amylopectin^63^. The peaks at 3725 cm^– 1^, 1751 cm^– 1^, 1648 cm^– 1^, and 1587 cm^– 1^ related to N–H, C = O of SMA, amide I, and amide II which are found in PNIPAm^18,64,65^. The amylopectin-S, N–CQDs15 showed the same peaks like the amylopectin-S, N–CQDs0 with additional peaks at 844 cm^– 1^ and 615 cm^– 1^ related to C–N and C–S od the incorporated S, N–CQDs within amylopectin-S, N–CQDs15. Additionally, the O–H group of amylopectin-S, N–CQDs15 film was shifted from 3442 cm^−1^ to 3397 cm^– 1^, which means the strong H-bonding in amylopectin-S, N–CQDs15 compared to amylopectin-S, N–CQDs0. The calculated MHBS (i.e., 0.97 and 0.99) and LOI (i.e. 0.97 and 0.98) for amylopectin-S, N–CQDs0 and amylopectin-S, N–CQDs15, respectively, which means high H-bonding strength between amylopectin-S, N–CQDs15 compared to amylopectin-S, N–CQDs0.

### Morphological observations

**Fig. 3:**
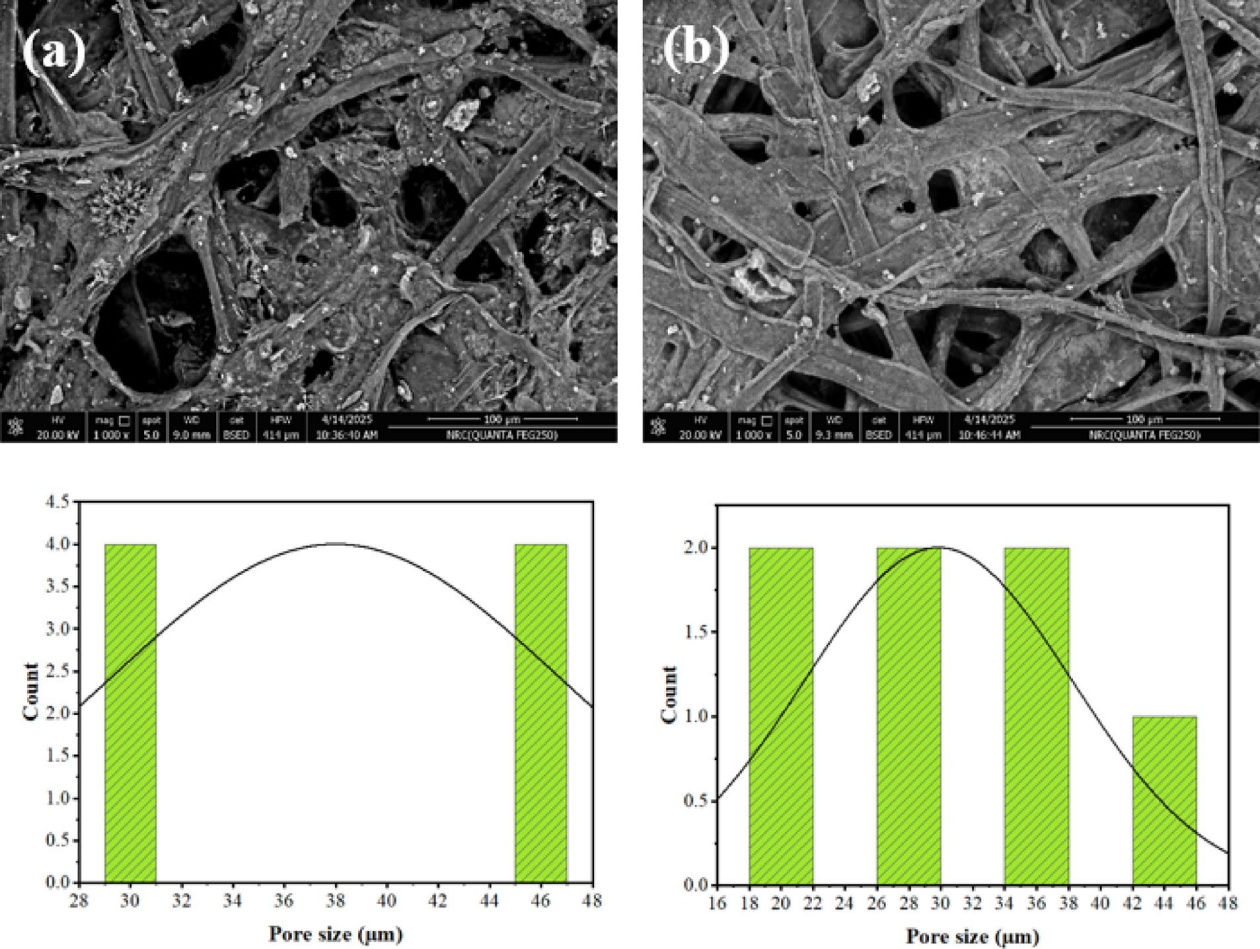
SEM analysis of (**a**) amylopectin-S, N–CQDs0 and (**b**) amylopectin-S, N–CQDs15 with pore size distribution.

Figure 3 shows that amylopectin-S, N–CQDs0 has larger pores (29.37–46.09 μm) compared to the more tightly packed structure of amylopectin-S, N–CQDs15, which exhibits pores ranging from 19.00 to 41.45 μm. The smaller pore size observed in the amylopectin-S, N–CQDs15, a direct consequence of incorporating S, N–CQDs, offers significant advantages for sensing applications when contrasted with the larger pores found in amylopectin-S, N–CQDs0, which lacks these S, N–CQDs. One key benefit arises from the substantial increase in surface area associated with a network of smaller pores. This expanded surface provides a greater number of active sites where analyte molecules can interact with the sensor material. Consequently, this enhanced interaction can lead to a more robust and sensitive sensor response, as more analyte molecules are available to trigger the sensing mechanism. Furthermore, the tighter spatial constraints within the smaller pores of amylopectin-S, N–CQDs15 can promote more efficient adsorption of analyte molecules onto the sensor surface, particularly crucial when the sensing mechanism relies on the binding of the analyte to the material. The close proximity of the pore walls in this structure increases the likelihood of these interactions occurring.

Importantly, the S, N–CQDs are not merely structural components; they can act as the primary active sensing elements due to their unique electronic and chemical properties. The smaller pore environment in amylopectin-S, N–CQDs15 can then serve to concentrate the analyte molecules in close proximity to these S, N–CQDs, effectively amplifying the resulting sensing signal. Additionally, the specific surface chemistry and electronic characteristics of the S, N–CQDs, in conjunction with the controlled spatial confinement provided by the smaller pores, may also contribute to improved selectivity towards target analytes by favoring specific interactions within the defined pore space. In essence, the smaller pore size in the S, N–CQDs-containing sensor, driven by the presence of these functional nanoparticles, is a key factor in achieving enhanced sensitivity and potentially improved selectivity compared to the sensor lacking these components and exhibiting larger pores.

### Antibacterial activity and molecular docking study

#### Antibacterial activity and mechanism of action

The amylopectin-S, N–CQDs xerogels demonstrated significant antimicrobial efficacy against key spoilage microorganisms. The amylopectin-S, N–CQDs15 xerogel, which contains 15% of S, N–CQDs, exhibited substantially higher antibacterial activity than the control film (amylopectin-S, N–CQDs0). Specifically, the amylopectin-S, N–CQDs15 film showed inhibition rates of 95.25% against *Escherichia coli*, 99.12% against *Staphylococcus aureus*, and 99.23% against *Candida Albicans*. In contrast, the control xerogel (i.e. amylopectin-S, N–CQDs0 xerogel) showed inhibition rates of 76.75%, 86.42%, and 76.20%, respectively.

#### Mechanism of action

The enhanced antimicrobial activity of the S, N–CQDs is attributed to a multi-pronged attack on the microbial cells. The mechanism involves both direct physical-chemical interactions and the induction of oxidative stress.

##### Direct interaction

The functional groups on the S, N–CQDs surface (such as amine and hydroxyl groups) engage in powerful interactions with essential microbial components. These include hydrogen bonding, π-π stacking, and electrostatic forces that allow the nanoparticles to bind to and disrupt the cell membrane, proteins, and even nucleic acids. This strong binding is supported by molecular docking studies, which showed that the amylopectin-S, N–CQDs15 composite formed shorter bond lengths with target proteins (2.52 Å for *E. coli*, 2.46 Å for *S. aureus*, and 2.02 Å for *C. albicans*) compared to the control. These shorter bond lengths indicate a stronger binding affinity, leading to greater disruption of microbial function and protein denaturation^3,4,66^.

##### Oxidative stress

S, N–CQDs have the ability to generate reactive oxygen species (ROS), such as superoxide radicals and hydrogen peroxide, when they interact with the microbial surface. These highly reactive molecules cause significant oxidative damage to the microbial cell, targeting critical components like the cell membrane and proteins. This widespread damage compromises the integrity of the cell, leading to its eventual death. This dual mechanism of physical disruption and chemical stress provides a robust^1,2,18,50^.

**Fig. 4:**
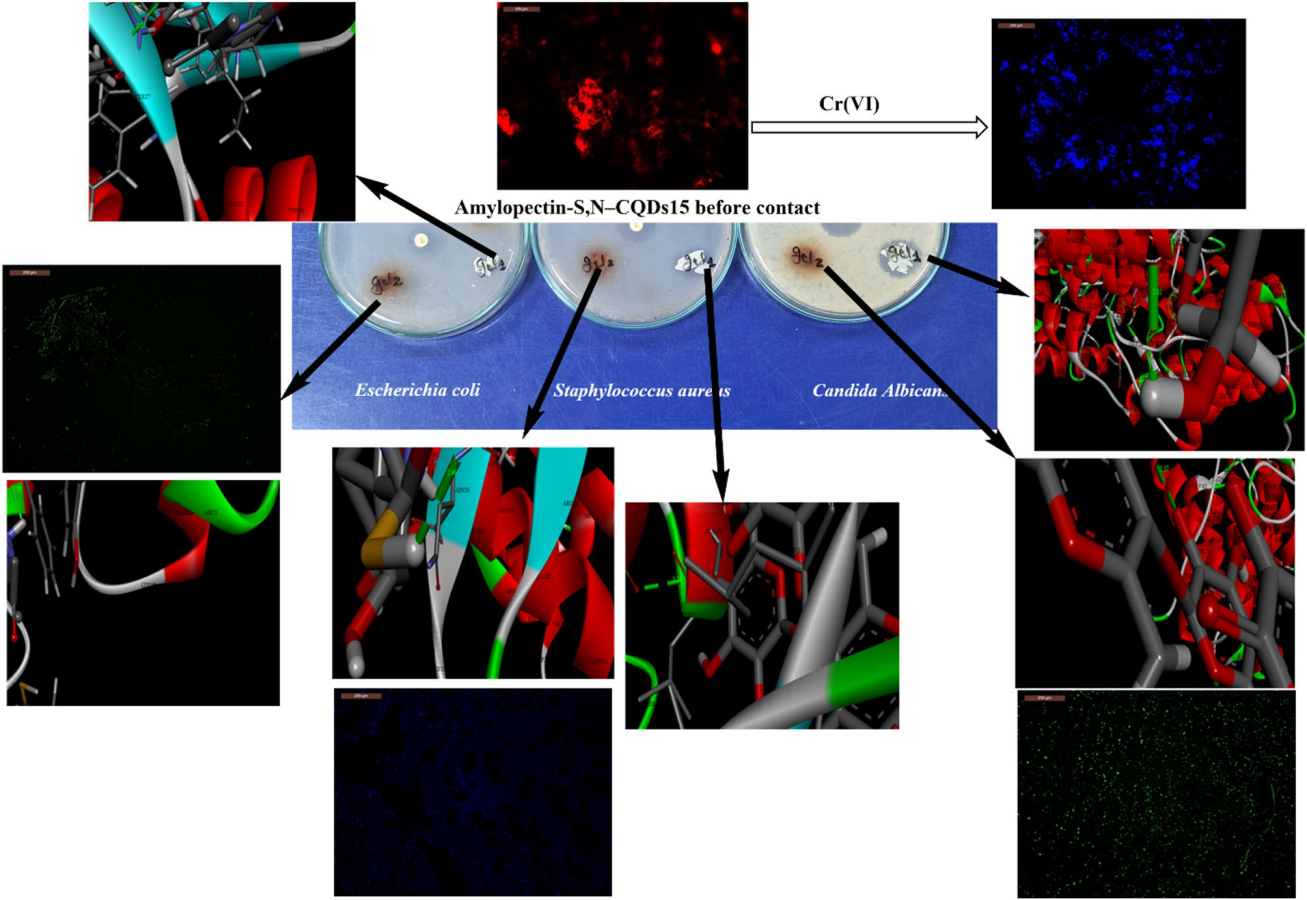
Antimicrobial activity of amylopectin-S, N–CQDs0 (denoted as gel 2) and amylopectin-S, N–CQDs15 (denoted as gel 1) against *Escherichia coli* & *Staphylococcus aureus* & *Candida Albicans*; molecular docking study towards *Escherichia coli* PDB (2M46), *Staphylococcus aureus* PDB (4QLO), *Candida Albicans* PDB (4YDE); and fluorescence microscope for amylopectin-S, N–CQDs15 (denoted as gel 2) before bacterial contact, after contact with *Escherichia coli*, after contact with *Staphylococcus aureus*, after contact with *Candida Albicans*, and after contact with Cr(VI).

### amylopectin-S, N–CQDs15 as a probe for detecting chromium, fungi and bacteria

The fluorescent properties of amylopectin-S, N–CQDs15 were confirmed by the observation of strong red fluorescence before exposure to bacteria, fungi, and Cr(VI) (Fig. 4). To assess the ability of the amylopectin-S, N–CQDs15 composite to identify bacteria and fungi; *Escherichia coli*, *Staphylococcus aureus*, and *Candida albicans* were used as representative pathogens in this study. These microorganisms were collected during their logarithmic growth phase for confocal microscopy experiments. After incubation with amylopectin-S, N–CQDs15, the resulting cell-conjugates were washed with ultrapure water and visualized via fluorescence microscopy. Distinct fluorescence patterns were evident upon interaction with each target microorganism. Specifically, when *Escherichia coli* was present, amylopectin-S, N–CQDs15 showed mixed green and smaller red fluorescent regions (Fig. 4). The Gram-negative bacterium *Escherichia coli* possesses an outer membrane rich in lipopolysaccharide (LPS), a crucial factor for its survival and pathogenicity in various environments. This LPS-rich outer membrane acts as a barrier, limiting direct contact between the S, N–CQDs and the bacterial cell wall. This steric hindrance caused by LPS modifies the S, N–CQDs’ fluorescence, resulting in the observed altered emission profile of mixed green and smaller red regions^1,2^. In the presence of *Staphylococcus aureus*, amylopectin-S, N–CQDs15 displayed blue dots (Fig. 4). This unique fluorescence profile is attributed to the higher density of negatively charged teichoic acids on the surface of *Staphylococcus aureus* cells compared to the LPS content in *Escherichia coli*. This greater negative surface charge promotes stronger electrostatic attraction and binding of the positively charged S, N–CQDs, yielding the observed blue fluorescence emission. The presence of oxygen, sulfur, and nitrogen functional groups from the poly(NIPAm) and the S, N-CQDs imparts a hydrophilic nature to amylopectin-S, N–CQDs15, which facilitates its efficient uptake into *Candida albicans* cells through endocytosis (Fig. 4)^49,50^. The change from large red regions to numerous green dots supports this cellular internalization, indicating successful intracellular localization^29^.

The shift in fluorescence from red to blue observed in amylopectin-S,N–CQDs15 upon Cr(VI) exposure is attributable to several underlying mechanisms (Fig. 4e). Firstly, Cr(VI) ions are known to be strong oxidizing agents and can interact with the surface of CQDs, leading to changes in their electronic structure^67,68^. This alteration can affect the energy levels within the CQDs, causing a shift in the emitted light’s wavelength^69^. Secondly, Cr(VI) can induce surface modifications on the CQDs, such as oxidation or the formation of new chemical bonds. These modifications can create new energy states, leading to a change in the fluorescence spectrum^67,70^. Thirdly, the interaction between Cr(VI) and the amylopectin-S,N-CQDs15 composite may involve the formation of complexes or adducts. These new chemical species can have different electronic transitions and thus emit light at different wavelengths compared to the original quantum dots. Finally, the change in the fluorescence color may also be attributed to inner filter effects (IFE), where Cr(VI) ions absorb either the excitation light or the emitted light from the CQDs^71,72^. However, for a color change from red to blue, this would imply that Cr(VI) absorbs red light more strongly than blue light, which is less common, but possible depending on the specific absorption spectrum of Cr(VI) in the solution and the emission spectra of the CQDs.

### pH-sensitivity of amylopectin-S, N–CQDs15 sensors for tomatoes spoilage by naked eye

**Fig. 5:**
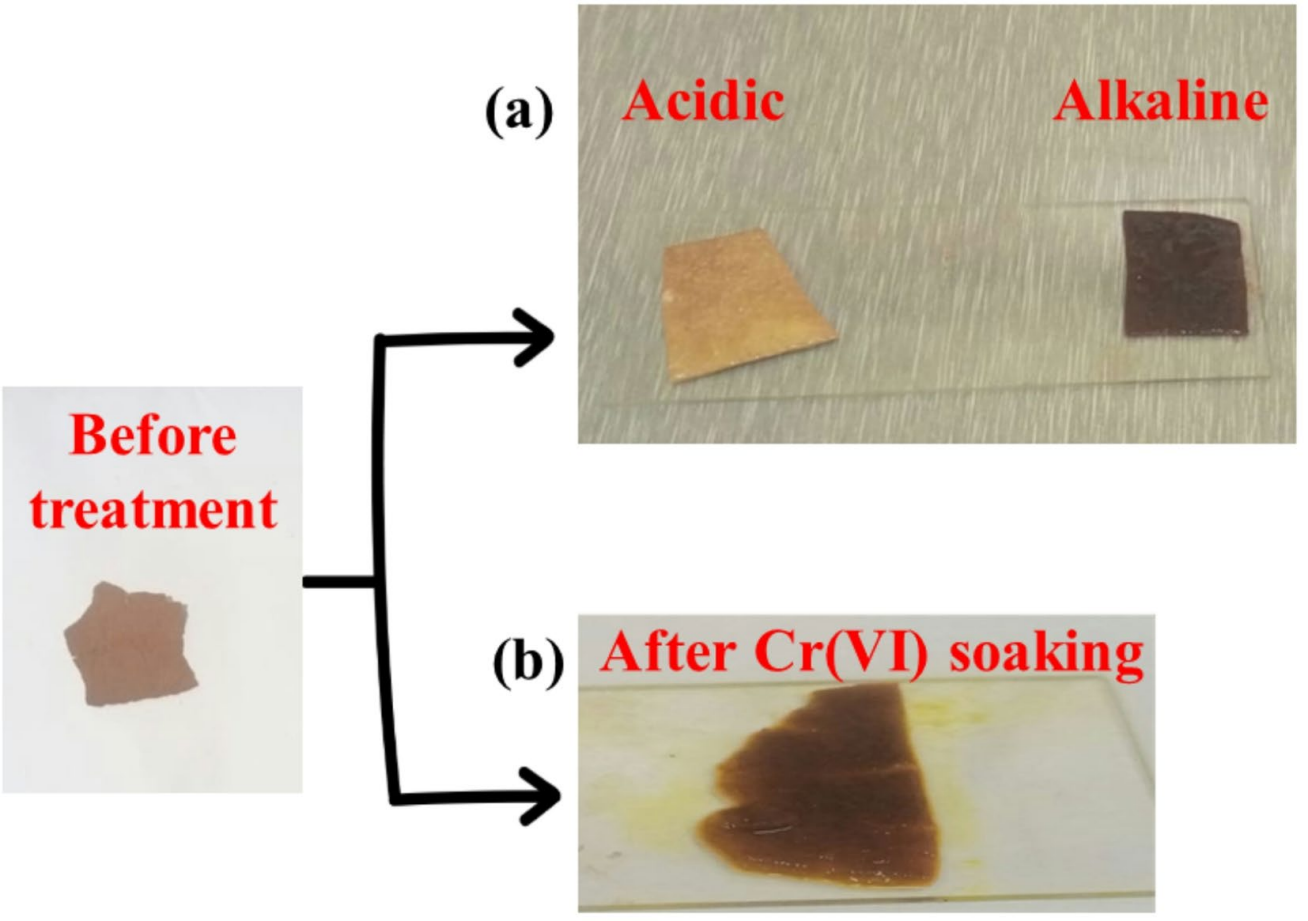

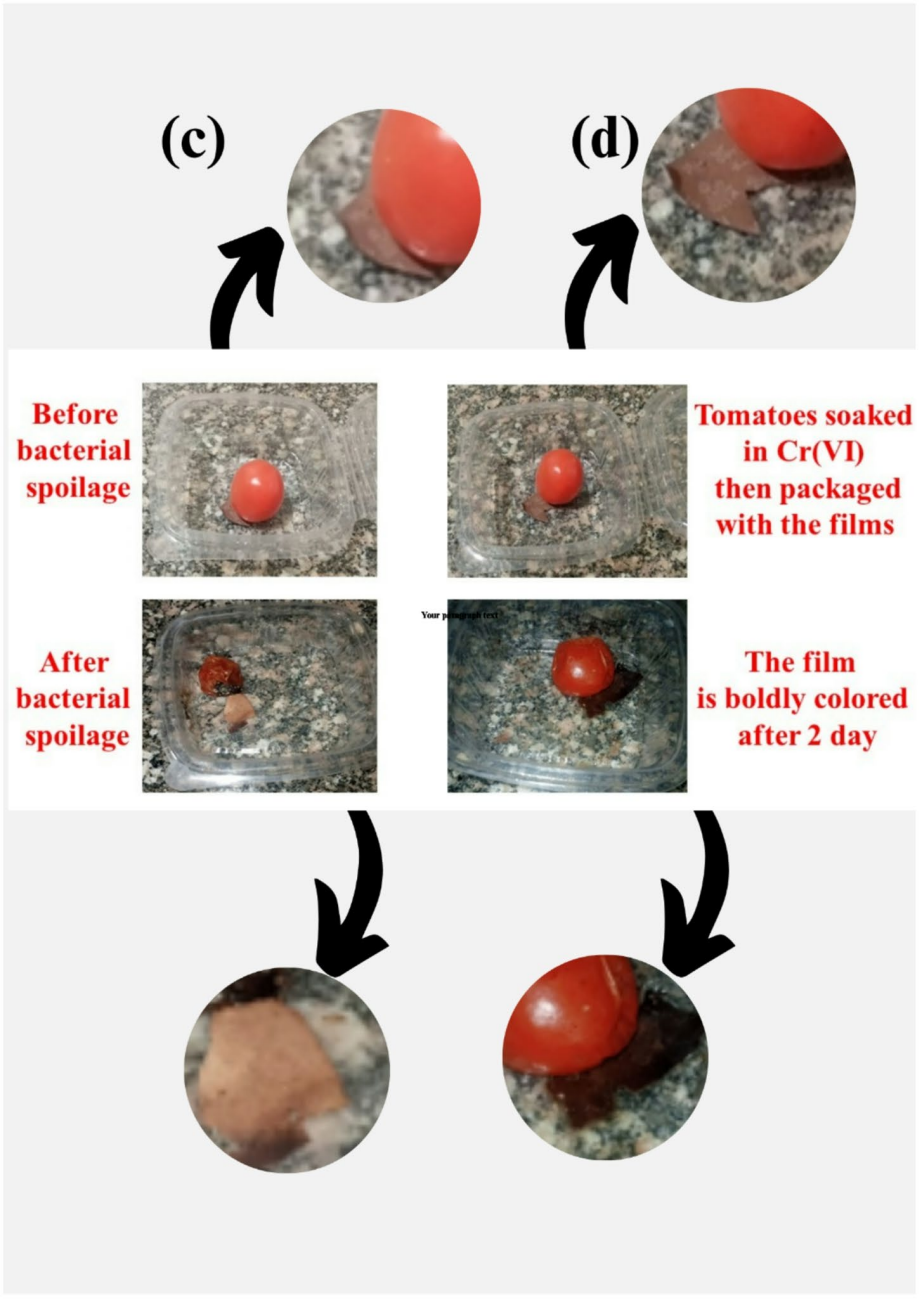
(**a**) Color response of amylopectin-S, N–CQDs15 at acidic and alkaline media, (**b**) Color response of amylopectin-S, N–CQDs15 after soaking in Cr(VI) solution, (**c**) Testing of amylopectin-S, N–CQDs15 hydrogel film on tomatoes spoilage by microorganisms, and (**d**) Testing of amylopectin-S, N–CQDs15 hydrogel film on tomatoes spoilage after Cr(VI) pollution.

The initial brown color suggests that the flavonoids were either in a different chemical state (perhaps influenced by a less acidic or alkaline environment during the initial observation) or interacting with other components of the film (i.e. Cr(VI)) in a way that modified their light absorption properties. As the film becomes more acidic, the protonation of the flavonoid molecules becomes more prevalent, leading to the expression of their characteristic yellow color under such conditions. The “fainter” aspect of the yellow might be attributed to extend the conjugated system which is leading to increased electron delocalization that slightly influence the final observed color. On contrary, the formation of phenolate ions, triggered by the loss of a proton from phenolic hydroxyl groups after alkaline treatment, dramatically reshapes the electronic structure of the molecule. This deprotonation breaks the continuous chain of alternating single and double carbon-carbon bonds (the conjugation system), which in turn modifies the way the molecule interacts with light. Consequently, the specific light absorption associated with these carbon-carbon bonds may weaken or disappear altogether due to these altered electronic transitions leading to the red color (Fig. 5a).

The intense color observed in the amylopectin-S, N–CQDs15 film upon exposure to Cr(VI), likely stems from fundamental differences in the chemical nature and interactions of the film components. Amylopectin, a branched polysaccharide, lacks the specific chromophoric structures present in flavonoids, which are responsible for their distinct colors and susceptibility to alteration through complexation or oxidation. The color of the amylopectin-based film is more likely inherent to the S, N–CQDs embedded within the matrix. These S, N–CQDs, with their unique electronic properties arising from their size, quantum confinement effects, and the presence of sulfur and nitrogen dopants, can exhibit strong fluorescence or absorbance in specific regions of the visible spectrum, leading to a bold and intense color (Fig. 5b). Furthermore, the interaction of Cr(VI) ions with the amylopectin-S, N–CQDs15 film would likely be different from their interaction with flavonoids. Amylopectin’s primary functional groups are hydroxyl groups, which may interact with Cr(VI) ions through weaker coordination or electrostatic interactions compared to the stronger complexation that can occur with the carbonyl and hydroxyl groups of flavonoids. Additionally, amylopectin itself is not as readily oxidized as flavonoids, which possess specific structural motifs that are susceptible to oxidative attack by strong oxidizing agents like hexavalent Cr(VI). Therefore, the Cr(VI) solution might not induce significant changes in the electronic structure or cause degradation of the color-producing S, N–CQDs within the amylopectin matrix, allowing the film to retain its intense coloration. The stability of the S, N–CQDs within the amylopectin matrix against the Cr(VI) solution is a key factor in maintaining the bold color, highlighting the influence of the film’s composition on its interaction with external chemical species.

The accompanying decrease in pH to approximately 5.2 during spoilage is a consequence of the metabolic byproducts produced by these microorganisms^1^. Many bacteria and fungi generate organic acids, such as lactic acid, acetic acid, and butyric acid, as end products of their fermentation processes. The accumulation of these acidic compounds lowers the overall pH of the tomato flesh^73^. This pH shift further contributes to the degradation of the fruit’s structural integrity and influences the activity of various enzymes, ultimately impacting the tomato’s quality, texture (loss of firmness due to cell wall breakdown), and flavor (spoilage)^74,75^. The observed color change of the amylopectin-S, N–CQDs15 hydrogel film from brown to yellow upon contact with spoiling tomatoes (as depicted in Fig. 5c) likely arises from the interaction of the film’s components with the metabolic byproducts released during the microbial spoilage process of tomatoes. The S, N–CQDs within the amylopectin matrix possess surface functional groups that can be sensitive to changes in their chemical environment. The acidic environment produced by the spoilage microorganisms can induce alterations in the electronic transitions within the S, N–CQDs. These changes in electronic structure can lead to a shift in the wavelengths of light absorbed or emitted by the S, N–CQDs, resulting in the observed color transition from brown to yellow. Additionally, the amylopectin matrix itself might undergo some chemical modification due to the acidic environment or enzymatic activity, potentially contributing to the overall color change of the film. The specific nature of the color change suggests a selective interaction between the film’s components and certain spoilage-related compounds. However, a contrasting observation was made when tomatoes contaminated with Cr(VI) were enclosed in the amylopectin-S, N–CQDs15 hydrogel film. Instead of fading, the film exhibited a rapid and pronounced intensification of color, shifting from its initial brown to a deeply saturated reddish color within approximately two days, with the increase in color intensity becoming noticeable within the same timeframe (as shown in Fig. 5d). This suggests a direct chemical reaction between the Cr(VI) present in the amylopectin-S, N–CQDs15 film and certain constituents of the tomato, leading to this significant color transformation of the film.

### Challenges and limitations of amylopectin-S, N–CQDs15 sensors

The primary limitation stems from the xerogel nature of the sensor is that their lack of mechanical strength due to the xerogel nature. As a xerogel, the material is formed by drying a hydrogel, which results in a brittle, rigid structure with limited mechanical flexibility^76,77^. This lack of elasticity makes the film prone to cracking or breaking upon handling, a significant drawback for its practical application as a flexible packaging film. A brittle sensor may not conform well to the shape of various food products and could fracture during shipping or storage, compromising its functionality.

## Conclusions

This study successfully demonstrates the development of a multifunctional, biodegradable food packaging film by incorporating S, N–CQDs derived from red onion peels into an amylopectin xerogel. The resulting amylopectin-S, N–CQDs15 film is a significant step forward in active food packaging technology, offering a synergistic combination of structural integrity, enhanced antimicrobial properties, and dual sensing capabilities. Our findings confirm that the incorporation of S, N–CQDs creates a more stable, compact structure with a larger surface area, directly contributing to the film’s improved performance. This is evidenced by the film’s remarkable antimicrobial efficacy, which achieved 95.25% inhibition against *E. coli* and over 99% against *S. aureus* and *C. albicans*. The xerogel film also functioned as a versatile visual biosensor, exhibiting distinct fluorescent responses to these pathogens and a clear colorimetric shift upon detecting toxic Cr(VI). These features, combined with its pH-responsive color change for general spoilage, make it a powerful tool for monitoring food freshness and safety in real-time. The application of this system on a large scale is highly plausible. The use of amylopectin, a readily available biopolymer, and S, N–CQDs synthesized from red onion peel waste makes the production process cost-effective and sustainable. The sensory outputs are visible to the naked eye or can be detected with simple instruments, allowing for rapid, on-site quality control without the need for complex laboratory analysis.

## Data Availability

Data will be made available on request from H.-A.S.T.
